# A Comparison Between Cardiac Telerehabilitation Program and Outpatient Hospital-Based Cardiac Rehabilitation Program for Patients Undergoing Coronary Artery Bypass Graft (CABG) Surgery: A Review

**DOI:** 10.7759/cureus.48488

**Published:** 2023-11-08

**Authors:** Saurabh N Puri, Lajwanti Lalwani

**Affiliations:** 1 Department of Cardiorespiratory Physiotherapy, Ravi Nair Physiotherapy College, Datta Meghe Institute of Higher Education And Research (DU), Wardha, IND

**Keywords:** coronary artery diseases, coronary artery bypass grafting(cabg), rehabilitation, cabg, cardiac telerehabilitation

## Abstract

Coronary artery disease (CAD) is a prevalent and possibly fatal cardiovascular ailment, and it is treated surgically by coronary artery bypass grafting (CABG). The coronary arteries, which supply the heart with oxygen and nutrients, are the most commonly affected. Even though CABG is a frequently employed procedure to restore cardiac blood flow, prolonged rehabilitation is necessary for long-term success. For patients with heart disease, cardiac rehabilitation (CR) involves a comprehensive therapeutic approach. It consists of risk mitigation, regular exercise, health education, and efficient stress management. Information and communication technology is used in telerehabilitation (TR), a rehabilitation service that offers a flexible choice that improves self-management. This study examined novel approaches and effective intervention elements while comparing cardiac TR with center-based programs in terms of risk factor management, patient commitment, and satisfaction.

## Introduction and background

Coronary artery bypass grafting (CABG), often called cardiac bypass surgery or bypass surgery, is one of the treatment options for coronary artery disease (CAD). CAD is caused by the accumulation of plaque, or atherosclerosis, in the coronary arteries, which are essential for supplying blood and oxygen to the heart muscle. This restriction may reduce blood flow to the heart, increasing the risk of a heart attack and perhaps producing angina (chest pain) [1]. CABG surgery is a high-risk procedure, and complications frequently necessitate emergency treatment. Multiple arterial blockages, recurrent angina, triple-vessel disease, and failed non-surgical therapies are all indications. The morbidity and mortality rates after 30 days might reach 14.0% and 2.0%, respectively.

Cardiac rehabilitation (CR) is a vital program that aids in the recovery and improvement of cardiovascular health in those suffering from heart disease. It not only helps with physical rehabilitation but also provides patients with the knowledge, psychological support, and lifestyle changes they need to take charge of their heart health. By addressing these critical factors, CR enables people to live healthier, longer lives while lowering their risk of future cardiac problems [2].

Following CABG, patients frequently attend CR, progressing through phases I-III of recovery. In the hospital, phase I consists of vital sign monitoring, pain management, movement, psychological support, and education. Phase 2 entails an outpatient program (center-based and home-based) that focuses on cardiovascular fitness, vital sign monitoring, dietary advice, psychological support, and lifestyle changes such as quitting smoking and losing weight. Telerehabilitation (TR) has been used in home-based cardiac rehab, which was initially intended as a telephone-based phase 2 CR [3]. During the coronavirus disease 2019 (COVID-19) pandemic, telemedicine, including TR, received considerable attention and was widely employed. Due to a lack of outpatient CR programs (centers) and COVID-related concerns, more patients were referred to our telemedicine CR, particularly following cardiothoracic surgery [4]. The development of rehabilitation efforts aimed at encouraging lifestyle modifications and self-care in cardiovascular diseases (CVD) is critical to lowering mortality rates in these patients. Successful rehabilitation is associated with fewer symptoms, a higher quality of life, less psychological distress, and a lower mortality rate [5].

TR is a growing subset of telehealth, which uses information and communication technology to provide healthcare services [6]. Hence, TR is defined as the provision of rehabilitation services via the use of information and communication technology [7]. TR could prove to be more compatible with the patient's way of life and improve self-management [8]. Patients who undergo CR have lower hospitalization and death rates [9]. Most CR programs are center-based, with patients working in groups with a physiotherapist [10]. Previous studies have demonstrated that, even in the absence of technology, home-based CR is just as effective as center-based programs in enhancing patients' quality of life and capacity for exercise [11]. Telemedicine can be regarded as a solution for some patients, according to guidelines for its use in CR [12]. This study aims to shed light on the technology used in these initiatives, as well as the features of the most effective interventions. Because their application in CR is still being researched, video gaming and virtual reality (VR) will not be assessed as part of digital health therapies in this study [13].

The results of this investigation showed that modifiable cardiac risk factors such as blood pressure, cholesterol, and BMI significantly decreased with both cardiac TR and center-based CR. Patients enrolled in cardiac TR had similar levels of satisfaction and attendance as those enrolled in typical center-based CR programs. This shows that cardiac TR can be equally successful in risk factor management as center-based CR while offering the advantage of remote access, which is especially helpful for patients with restricted mobility or those who reside in remote areas.

## Review

Sources of information and search process

For this study, we included original publications, systematic reviews, meta-analyses, and randomized controlled trials (RCTs). Keywords and Medical Subject Headings (MESH) phrases were used to find the publications. The papers were also screened using keywords. The papers were chosen using the keywords "cardiac rehabilitation and telerehabilitation, outpatient rehabilitation, center-based rehabilitation, cardiovascular disease, CABG, and cardiac rehabilitation". The databases PubMed, Scopus, Web of Science, and Google Scholar were used to find articles.

Methodology

**Figure 1:**
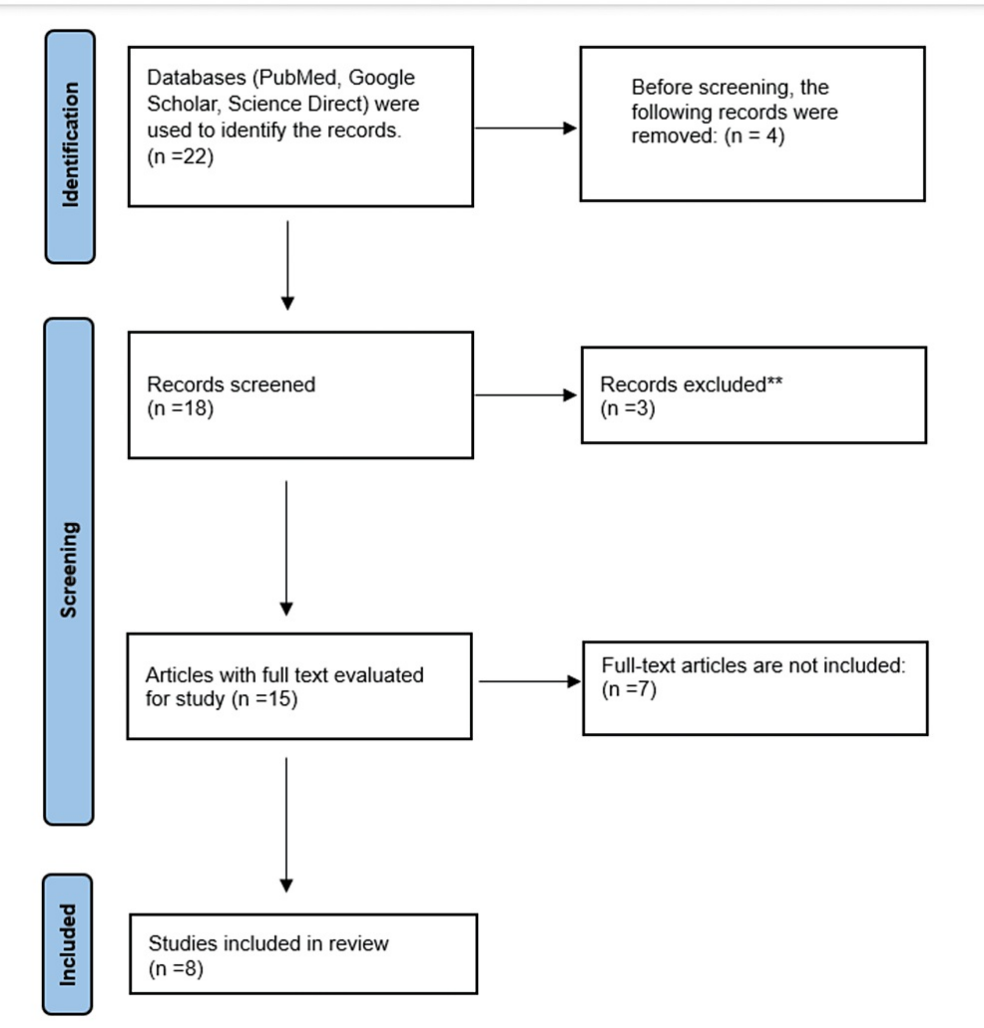
Chart depicting the selection of articles

A total of 22 records of systematic literature were initially examined for eligibility as full-text publications. After removing unrelated studies and duplicates and applying other criteria for exclusion, this review ultimately included a total of eight papers. One of the key reasons for exclusion was the inability to access the full version of articles. As a result, additional literature on the subject would be required. There were several types of studies, including experimental studies, RCTs, systematic reviews, and literature reviews. Figure 1 depicts the article selection process in compliance with the PRISMA guidelines.

Coronary artery bypass grafting (CABG)

CABG refers to a surgical treatment that is used to treat CAD. CABG involves grafting or connecting blood vessels (usually arteries from the chest or veins from the leg) surrounding obstructed or restricted coronary arteries to create new channels for blood to circulate to the heart muscle. This operation is used to enhance the function of the heart muscle by restoring enough blood flow. CABG has been in use for almost 50 years. Additionally, the surgery is now being performed more frequently on elderly and higher-risk patients [14]. CABG is advised for severe CAD, unresponsive angina, left main coronary disease, triple-vessel disease, failed non-surgical therapies, and complicated coronary difficulties. It is also useful in diabetic crises and heart attack emergencies and is very effective for severe angina [15]. When medication or percutaneous transluminal coronary angioplasty (PTCA) fails to relieve angina, CABG is chosen, especially if it can delay unfavorable outcomes such as death, heart attacks, or angina recurrence more successfully than other therapies [16]. In the early years following surgery, patients with symptomatic left major coronary artery stenosis or stenosis of the three main coronary arteries had a better prognosis following CABG, even though this advantage is not expected to last for more than 10-12 years [15]. While CABG is a highly effective treatment for many people with CAD, it does have certain limitations and potential risks. These include its invasive nature as it is a major surgical procedure, longer recovery time, and limited graft longevity; also, CABG carries risks such as infection, bleeding, stroke, heart rhythm disturbances, and, in rare cases, mortality. Findings from the obtained studies offer valuable insights into the efficacy and concerns associated with CABG as a CAD therapy technique.

Cardiac rehabilitation

CR programs focus on physical activity, risk factor education, lifestyle changes, dietary counseling, and medication optimization. These factors reduce physiological and psychological stress, which enhances the quality of life and reduces the risk of cardiovascular disease [16]. CR treatments, which include patient education, health behavior modification techniques, and exercise training, all enhance clinical results when used for heart disease [17]. CR is divided into three stages: phase I in the hospital, phase II as an outpatient program lasting up to week 36 after a cardiovascular event, and phase III/IV for long-term maintenance. Phase II focuses on supervised physical activity that is tailored to the findings of individual clinical exams and exercise stress testing [18]. Before starting a training program for a physiologically thorough exercise intensity prescription, it is imperative to perform a functional evaluation through exercise testing. An important problem related to both the rate of improvement (CR efficacy) and the possibility of unfavorable outcomes while exercising is the prescription of exercise intensity [19]. Phase III/IV aims to stabilize the patient's health by continuing with the established lifestyle modifications with little to no professional monitoring [18]. While CR criteria vary, they often include those who have experienced a cardiac event (such as a heart attack), heart surgery (such as CABG), or other heart-related treatments. Patients with stable angina, heart failure, or heart valve surgery may also be eligible. High-risk variables are often investigated, including diabetes, obesity, high cholesterol, and hypertension. A physician's reference is required for several programs.

Center-based CR strategies at hospitals, gymnasiums, clinics, healthcare centers, and athletic venues help cardiac patients increase their quality-adjusted life years [20]. Remote patients benefit from home-based cardiovascular rehab that uses technology to provide personalized treatment. Telemedicine reaches out to new groups by increasing home-based cardiac rehab programs [21]. Clinical studies show that both center-based and home-based CR programs improve the lifestyle and well-being of individuals who have had a heart attack or revascularization [22]. CR may be necessary following an acute myocardial infarction, coronary artery bypass surgery, heart valve replacement or repair, or for those with systolic heart failure or chronic stable angina [23]. TR and mobile technologies are emerging as options to overcome limited participation. TR provides feedback, coaching, and specialized counsel over the Internet, phone, or videoconferencing to extend home-based cardiac rehab [24]. TR enables people to receive CR without having to visit hospitals or healthcare institutions. They might instead finish the rehabilitation regimen from the comfort of their own home.

Treatment

Cardiovascular rehab is usually classified into three stages. The goals of each stage of cardiac CR are to aid in healing and avert further cardiovascular illness [25]. For diagnostic and therapeutic purposes, a symptom-limited test is conducted before exercise training. Both programs stress the need to incorporate endurance and resistance training by using a range of equipment in exercise routines. The key distinction lies in how the exercise prescription is tailored. Patients with heart failure and stable CAD are recommended to participate in both programs high-intensity interval aerobic exercise [26,27]. Increased daily activity should be combined with aerobic exercise, such as brisk walking. CR includes dietary counseling, weight management, blood pressure control, quitting smoking, and diabetes management. TR overcomes geographical barriers by allowing patients who are unable to go to localized CR centers to interact remotely [28]. For CAD patients, TR may have long-term benefits [29]. TR in heart failure (HF) can minimize unplanned hospitalizations and overall mortality [30]. TR has a better chance of having a lifelong impact than center-based CR [31].

Discussion

Cardiovascular rehabilitation programs that focus on telemedicine have the potential to enhance the long-term health results of patients who are stationed distant [32]. The primary conclusions of the study are that for patients who can benefit from technology, cardiac TR is just as successful as standard center-based programs and can be delivered utilizing a range of technologies. Phase III therapies with motivating feedback, self-monitoring, or instructional components were often the interventions that proved to be most successful at controlling patient's functional capacity and physical activity [33]. There are various studies in the literature regarding CR for CAD, as summarized in Table 1.

**Table 1:** Various studies regarding cardiac rehabilitation for coronary artery disease RCT: randomized controlled trial; CV: cardiovascular; CR: cardiac rehabilitation; ACS: acute coronary syndrome; CABG: coronary artery bypass graft; CAD: coronary artery disease; PCI: percutaneous coronary intervention VO_2_peak: peak oxygen uptake: DHI: digital health intervention

Author and year	Design	Sample size	Intervention and outcome measure	Conclusion
Poortaghi et al. (2013) [34]	RCT	80 people with CAD took part in the study	The therapeutic benefit of interdisciplinary home-based CR vs. center-based CR	A home-based rehabilitation program improves patients' self-efficacy. These findings further demonstrate that adequate and effective patient education, continuity of treatment, and home follow-up can alleviate the challenges posed by patients who are not referred
Bravo-Escobar et al. (2017) [35]	RCT	28 people with moderate CAD and a cardiovascular risk factor took part in the study	The comparison of home-based CR with hospital-based (mixed surveillance) CR in patients with ischemic heart disease; functional capacity, blood pressure, cholesterol, BMI, WC, and HRQoL are all measured	Patients with ischemic heart disease benefitted greatly from home-based and hospital-based CR programs in terms of quality of life and recovery rate
Frederix et al. (2017) [36]	Randomized controlled telerehabilitation trial	140 participants with CAD and/or CHD who had completed half of a 12-week center-based CR regimen	Following 12 weeks of regular center-based CR, patients received 6 months of cardiac telerehabilitation. Patients were given individualized exercise prescriptions and the opportunity for self-monitoring. Every week, patients input data into a web application and got educational materials, comments, and recommendations	A combination of telerehabilitation and center-based therapy, followed by transitional telerehabilitation, produced long-term health advantages and was affordable for up to two years following the intervention
Skobel et al. (2017) [37]	RCT	132 individuals with CAD who had also had an MI or an acute coronary intervention. Patients had completed a phase II CR course before the commencement of the trial	Patients got standard therapy as well as 6-month access to a web application that included exercise prescriptions and remote monitoring. The application supplied educational content. Functional capacity, BP, lipids, BMI, anxiety, depression, and HRQoL were observed	There were no training-related issues. Peak VO_2_ improved greater in individuals who finished the research. A newly built home-based CR system looks practicable, and safe, and enhances exercise capacity
Widmer et al. (2017) [38]	RCT	80 patients who have had PCI for ACS	12-week phase II CR at a center, including access to a digital health intervention. Patients were given instructional materials and automated guidance after uploading information about their activity and food to a web application.	The study found that supplementary DHI increases weight reduction considerably and may offer a technique to prevent CV-related emergency department visits and rehospitalizations in patients undergoing CR following ACS. The study implies that DHI might be used in addition to CR to improve secondary CV disease prevention
Kraal et al. (2017) [39]	RCT	90 patients were admitted to CR following an ACS or revascularisation operation	12-week fitness routine at home. Patients uploaded data using a chest HR monitor and a web interface. Physiotherapists called patients once a week to provide motivational comments.	According to this study, home-based CR is equivalent to center-based CR in terms of enhancing fitness and quality of life. Home-based CR resulted in higher exercise adherence and higher patient satisfaction. It can boost exercise participation, especially for people who need to go back to work soon or who have transportation concerns. It also looks to be cost-effective, making it a viable option for motivated low-to-moderate cardiac risk patients beginning CR
Avila et al. (2018) [40]	RCT	90 people with CAD took part in the study	The ability of telemonitoring-focused, at-home cardiac rehabilitation to help individuals with coronary artery disease become more physically fit was evaluated.	Following the completion of the phase II ambulatory CR program, adding a home-based exercise program with telemonitoring instruction leads to additional development of physical fitness and is as beneficial as continuing a center-based CR in patients with CAD
Spindler et al. (2019) [33]	RCT	151 participants with HF, arterial stenosis, previous CABG, or valve surgery	12-week phase II CR regimen at home. Patients uploaded self-monitoring data and received instructional materials via a web application. Patients might use the app to communicate with healthcare providers.	The study found that while the telerehabilitation group showed an improvement in independent motivation at the beginning, this favorable variation in motivation did not last. As a result, it appears that neither rehabilitation technique can provide long-term motivation

## Conclusions

Cardiovascular TR, which is as effective as center-based programs, uses a variety of delivery techniques as well as thorough monitoring. According to research, patients who receive telemedicine assistance during phase III of CR have additional health benefits. In comparison to the other two therapies, our home-based exercise program was equally as beneficial and increased levels of ability to exercise or engage in physical activity. Home-based training is less costly than in-person instruction and has been linked with improved patient satisfaction. Several studies have also suggested that both combinations are also very effective. Finally, home-based training with telemonitoring support may be an option for center-based training for patients with low to moderate cardiac risk who are starting CR.
